# Enhancing prediction accuracy of foliar essential oil content, growth, and stem quality in *Eucalyptus globulus* using multi-trait deep learning models

**DOI:** 10.3389/fpls.2024.1451784

**Published:** 2024-10-10

**Authors:** Daniel Mieres-Castro, Carlos Maldonado, Freddy Mora-Poblete

**Affiliations:** ^1^ Laboratory of Genomics and Forestry Biotechnology, Institute of Biological Sciences, University of Talca, Talca, Chile; ^2^ Centro de Genómica y Bioinformática, Facultad de Ciencias, Universidad Mayor, Santiago, Chile

**Keywords:** *Eucalyptus* essential oil, wood production, deep learning, genomic prediction, phenomic prediction, multi-trait, multi-omic, high-throughput plant phenotyping and genotyping

## Abstract

*Eucalyptus globulus* Labill., is a recognized multipurpose tree, which stands out not only for the valuable qualities of its wood but also for the medicinal applications of the essential oil extracted from its leaves. In this study, we implemented an integrated strategy comprising genomic and phenomic approaches to predict foliar essential oil content, stem quality, and growth-related traits within a 9-year-old breeding population of *E. globulus*. The strategy involved evaluating Uni/Multi-trait deep learning (DL) models by incorporating genomic data related to single nucleotide polymorphisms (SNPs) and haplotypes, as well as the phenomic data from leaf near-infrared (NIR) spectroscopy. Our results showed that essential oil content (oil yield) ranged from 0.01 to 1.69% v/fw and had no significant correlation with any growth-related traits. This suggests that selection solely based on growth-related traits did n The emphases (colored text) from revisions were removed throughout the article. Confirm that this change is fine. ot influence the essential oil content. Genomic heritability estimates ranged from 0.25 (diameter at breast height (DBH) and oil yield) to 0.71 (DBH and stem straightness (ST)), while pedigree-based heritability exhibited a broader range, from 0.05 to 0.88. Notably, oil yield was found to be moderate to highly heritable, with genomic values ranging from 0.25 to 0.60, alongside a pedigree-based estimate of 0.48. The DL prediction models consistently achieved higher prediction accuracy (PA) values with a Multi-trait approach for most traits analyzed, including oil yield (0.699), tree height (0.772), DBH (0.745), slenderness coefficient (0.616), stem volume (0.757), and ST (0.764). The Uni-trait approach achieved superior PA values solely for branching quality (0.861). NIR spectral absorbance was the best omics data for CNN or MLP models with a Multi-trait approach. These results highlight considerable genetic variation within the *Eucalyptus* progeny trial, particularly regarding oil production. Our results contribute significantly to understanding omics-assisted deep learning models as a breeding strategy to improve growth-related traits and optimize essential oil production in this species.

## 1 Introduction

The *Eucalyptus* genus comprises more than 900 species and subspecies distributed in several environmental conditions, including arid, semi-arid, tropical, oceanic, and Mediterranean climates (Drake et al., 2015; Ballesta et al., 2018). Some *Eucalyptus* species are renowned for their remarkable biomass production, rapid growth rate, and exceptional adaptability (Mora et al., 2019; Ballesta et al., 2018; Ballesta et al., 2019). They have been cultivated across a global plantation area exceeding 22.57 million hectares (ha) worldwide, spanning over 90 countries with the major centers of cultivation in Brazil (5.7 million ha), India (3.9 million ha) and China (4.5 million ha) (FAO, 2020; Seng et al., 2022). *Eucalyptus* plantations serve as a valuable resource for the forestry industry, as they constitute the primary sources of biomass globally and among the main hardwoods utilized in pulp and wood production (Paiva et al., 2011; Mora et al., 2019; Ballesta et al., 2018, Ballesta et al., 2019). Additionally, several *Eucalyptus* species contain bioactive compounds, contributing to the production of diverse agro-based industrial products (Mieres-Castro et al., 2021). In fact, *Eucalyptus* compounds have diverse applications in nutraceuticals (Hamed et al., 2021), natural food preservatives (Kumar Tyagi et al., 2014; Boukhatem et al., 2020; Kheloul et al., 2023), pharmaceuticals (Salehi et al., 2019; Silveira et al., 2020; Chandorkar et al., 2021; Mieres-Castro et al., 2021), agricultural crop protection (Üstüner et al., 2018; Tomazoni et al., 2018; da Silva et al., 2020; Oli et al., 2019; Pedrotti et al., 2019, Pedrotti et al., 2020, Pedrotti et al., 2022), and renewable biofuels (Kainer et al., 2015, Kainer et al., 2017, Kainer et al., 2018, Kainer et al., 2019). Moreover, *Eucalyptus* terpene-based essential oils are economically important commodities (Barbieri and Borsotto, 2018; Kainer et al., 2019), which are frequently produced on an international scale as by-products in plantations of species such as *E. polybractea*, *E. smithii*, and *E. globulus*, primarily cultivated for their wood (Kainer et al., 2015, Kainer et al., 2017). The oil production and related traits in commercially harvested *Eucalyptus* species depend on complex quantitative factors, including foliar oil content, foliar biomass, and environmental adaptation (Kainer et al., 2015).

*Eucalyptus globulus* Labill is a key source of foliar essential oil used for pharmacological purposes, attributed to its elevated content of the main bioactive monoterpene, 1,8-cineole (commonly known as eucalyptol), which can comprise over 80% of the total oil (Mieres-Castro et al., 2021). Its bioactive compounds, including 1,8-cineole, contribute to pharmacological advancements and also hold potential for the development of eco-friendly natural products (Almeida et al., 2024). This distinctive species is also among the most widely cultivated hardwood trees in temperate regions of the world, prized for its application as raw material in the pulp and paper industry due to its high-quality cellulose pulp, along with low lignin and lipid content (Aumond et al., 2017; Ballesta et al., 2019; Mora et al., 2019). The tree’s adaptability and rapid growth make it a valuable asset for afforestation projects to mitigate environmental challenges (Ballesta et al., 2019; Mora et al., 2019). As a resilient and economically important species, *E. globulus* continues to play a pivotal role in ecological conservation efforts and various sectors of sustainable development, highlighting its multifaceted contributions to a more robust and sustainable global environment (Tomé et al., 2021).

The implementation of cutting-edge molecular approaches, exemplified by genotyping by sequencing and the utilization of high-density DNA arrays, has significantly propelled the field of genomic prediction (Ballesta et al., 2019). This progress is particularly notable in the application of several models to predict productivity traits in many crops and trees (Jung et al., 2022; Kent et al., 2023; Liao et al., 2022; Parveen et al., 2023). Alternatively, the canopy spectral reflectance and vegetation indices have been used as phenomics data to improve the prediction of genomic models (Ballesta et al., 2022). This is due to their ability to provide swift and affordable information on several traits of industrial interest in *Eucalyptus* and other species (Ballesta et al., 2022; Rincent et al., 2018). Recent advancements in the field of industrial crops research have emphasized the development and application of Multi-trait and/or Multi-environment genomic prediction models integrated with Machine Learning and Deep Learning methodologies, offering a promising solution for selective crop breeding (Maldonado et al., 2022). These models have demonstrated significant improvements in prediction accuracy (PA) over traditional models and Uni-trait approaches, especially in cases where traits have low or negative correlations (Mora-Poblete et al., 2023). Their efficacy becomes even more pronounced in predicting traits that are inherently challenging or expensive to phenotype within species of agro-industrial interest, as highlighted by recent studies (Maldonado et al., 2020, Maldonado et al., 2022; Mora-Poblete et al., 2023). To our best knowledge, no studies have applied Multi-trait and Multi-omics approaches, or an integrated phenomic/genomic method with artificial neural models, to predict phenotypic traits of industrial interest in *E. globulus*, highlighting a significant gap in research and breeding efforts (Rambolarimanana et al., 2018; Ballesta et al., 2018, Ballesta et al., 2019; Mora et al., 2019; Maldonado et al., 2022). Implementing these advanced methodologies could contribute to the development of genetically improved individuals and enhance the sustainability of essential oil production and related traits (Kainer et al., 2015, Kainer et al., 2017, Kainer et al., 2018, Kainer et al., 2019; Mazanec et al., 2021), which in turn supports the sustainable production and consumption of *E. globulus* across different industries, demonstrating the multifaceted benefits of integrating cutting-edge technologies into agro-industrial practices (Kainer et al., 2017; Boukhatem et al., 2020; Hamed et al., 2021; Khazraei et al., 2021; Pedrotti et al., 2022).

In response to these challenges and opportunities, this study aimed to improve the prediction accuracy of industrial phenotypic traits such as essential oil content, stem quality, and growth-related traits, in *E. globulus* by a Multi-trait and Multi-omics deep learning (DL) approach. This approach paves the way for advancements in sustainable agricultural and forestry practices. In this study, the DL models incorporated genomic data related to single nucleotide polymorphisms (SNPs) and haplotypes, as well as phenomic data from NIR spectral absorbance, to predict traits of industrial interest in a 9-year-old breeding population. The insights and findings presented in this study significantly contribute to advancing our understanding of breeding strategies based on omics-assisted deep learning models to improve traits of industrial interest in *E. globulus*, ultimately promoting progress in plant science and facilitating more effective and targeted breeding efforts.

## 2 Materials and methods

### 2.1 Plant material

The study’s breeding population of *Eucalyptus globulus* consisted of 62 full-sib and 3 half-sib families, totaling 1,968 individuals, which were selected for improving wood production-related traits. These families were sourced from forest seed orchards of Semillas Imperial SpA, Chile. The progeny trial was established in 2012 in La Poza, Purranque, in the administrative region of Los Lagos, Chile (40°58’S, 73°30’W, 326 m.a.s.l.). The prevailing climate in this area is an Oceanic or Marine climate type with an annual accumulated rainfall of 1282 mm and an average annual temperature of 13°C (Ballesta et al., 2018). The experimental design was a randomized complete block, with 30 blocks, single-tree plots, and a spacing of 2.5 m between the trees within a block (Ballesta et al., 2018, Ballesta et al., 2019; Mora et al., 2019).

### 2.2 High-throughput phenotyping and genotyping

The absolute reflectance of leaves (0.1 g lyophilized powder per individual) was measured following the methodology of Castillo et al. (2008) using a NIR spectrometer (NIRQuest512 spectrometer, Ocean Optics, Inc., Orlando, FL, USA), an HL-2000-HP-FHSA light source, and a 3.18 mm diameter bifurcated optical fiber (QR600-7-VIS- 125F). The NIR system was calibrated using a Spectralon^®^ reflectance standard (Labsphere, Inc., North Sutton, NH, USA). The measurements covered the spectral range from ~900 to 2500 nm. The equipment was set to integrate three samples per scan. The NIR spectral absorbance values were calculated as log(1/R) (where R is the reflectance spectra). The spectral data were pre-processed in the R 4.0.5 software (Core Development Team, 2020) following the method of Rincent et al. (2018), in which the spectral absorbance values were normalized (centered and scaled), and their first derivative was computed using a Savitzky–Golay filter (window size of 37 points).

Genomic DNA was extracted from the leaves of 339 randomly selected individuals (Ballesta et al., 2018). Genotyping of individuals was carried out using the EUChip60K SNPs system (GeneSeek, Lincoln, NE, USA). The genotyping quality of the samples was evaluated in Genome Studio software (Illumina, San Diego, CA). The genotyping quality of the samples was assessed using the Genome Studio software (Illumina, San Diego, CA). The SNPs with a minor allele frequency of<0.05 and a call rate of<90% were excluded from the data matrix, resulting in 14,442 high-quality SNPs for the individuals. Haplotype blocks were identified using a confidence interval algorithm in Haploview v. 4.2 (Ballesta et al., 2019). It was determined that two SNPs were in strong linkage disequilibrium (LD) if the coefficient of disequilibrium (D′) value was high (upper limit > 0.98 and lower limit ≥ 0.7). D′ values were calculated between loci A and B, and the physical positions of each SNP were determined based on the consensus map of the *Eucalyptus grandis* genome. Omics datasets, comprising phenomic data from NIR spectral absorbance and genomic data related to SNPs and haplotypes, were used to develop Uni/Multi-trait and Uni/Multi-omic deep learning models for predicting traits of industrial interest (as detailed in section 2.5).

### 2.3 Measurements of phenotypic traits

Phenotypic traits of industrial interest related to foliar essential oil content and wood production-related traits were assessed in 9-year-old trees. Fresh, fully expanded, mature leaves were collected from the northeastern side of the canopy to measure foliar essential oil content. The leaves were stored in airtight plastic bags at 4°C and transported under refrigeration to the laboratory, where they were immediately frozen at -20°C until processing. Foliar essential oils were extracted by hydrodistillation, following established protocols from previous studies with *E. globulus* (Zrira et al., 1992; Silvestre et al., 1997; Kassahun and Feleke, 2019; Ngo et al., 2020). Briefly, a total of 100 grams (g) of fresh leaves per individual were cleaned with distilled water and ground in a waring blender with 750 milliliters (mL) of distilled water. The essential oil from grounded fresh leaves was extracted at 100°C for 3 hours using a Clevenger-type apparatus, glassware, and standard instruments recommended in the European Pharmacopoeia (European Pharmacopoeia, 2020). The hydrodistillation process was carried out 3 times for each individual and the essential oil content was calculated as a percentage of oil yield (oil yield) using the following equation:


$$Oil\;yield\;=\;\left[\frac{Volume\;of\;essential\;oil\;\left(mL\right)}{Leaf\;fresh\;weight\;\left(g\right)}\right]\times 100\;$$

Wood production-related traits were assessed by measuring the following phenotypic attributes: tree height (TH), diameter at breast height (DBH), slenderness coefficient (SC), stem straightness (ST), branching quality (BQ), and stem volume (VOL). TH was measured using a hypsometer from ground level to the highest point of the tree. DBH was measured with a diameter tape at 1.3 m above ground level. SC was calculated according to Valenzuela et al. (2019), Valenzuela et al, 2021), with a SC = TH/DBH. ST, BQ, and VOL were evaluated according to Ballesta et al. (2018), Ballesta et al, 2019) and Mora et al. (2019).

**Supplementary Table S1** presents the compiled values from the measurement of industrial phenotypic traits of interest in *E. globulus* individuals. The relationship between the evaluated traits was analyzed by calculating the average Pearson correlation coefficient (between quantitative traits) and Spearman’s rank correlation coefficient (between categorical traits). Correlation tests were conducted using R 4.0.5 software (Core Development Team, 2020).

### 2.4 Genomic and pedigree-based heritability

In this study, the heritability estimates were based on both genetic data derived from an array of SNP markers and pedigree information. For heritability estimation based on the genomic information, the following models were used: Bayes A (Meuwissen et al., 2001), Bayes B (Meuwissen et al., 2001), Bayes C (Habier et al., 2011), and Bayesian Ridge Regression (BRR; Gianola, 2013) implemented in BGLR library (Pérez and de los Campos, 2014) in R 4.3.2 software (Core Development Team, 2020). These models were implemented according to Ballesta et al. (2020). On the other hand, in heritability estimation based on a pedigree model, individual breeding values were estimated using a Bayesian generalized linear model implemented through the MCMCglmm library (Hadfield, 2010) in R 4.3.2 software (Core Development Team, 2020) according to Mora et al. (2019).

### 2.5 Uni/multi-trait and uni/multi-omic deep learning models

#### 2.5.1 Convolutional neural networks and multilayer perceptron

The CNN was implemented following the methodology proposed by Pérez-Enciso and Zingaretti (2019), utilizing a convolutional layer (conv1D) for effective feature extraction. The layers of this approach follow a hierarchical structure, which has a tremendous capability of extracting robust features at each of the layers through the learning process (Maldonado et al., 2022). Briefly, the architecture was composed of (I) an input layer for loading the input data with n (number of molecular markers or spectral signatures) neurons, (II) two Conv1D layers for feature extraction from the molecular markers or spectral data (considering a kernel matrix or weight matrix), (III) 1D max pooling layer (Maxpool1D) for reduces the resolution to dividing the input into 1D pooling regions and computing the maximum value of the feature map in each region, (IV) flatten layer for creating a one-dimensional vector through flatten the input data, (V) two dense layers (fully connected layer), which implies that the neurons between this layer and its preceding layer are fully connected, and (VI) output layer (dense layer for prediction) which employs the linear activation function for prediction problem. The MLP was implemented according to Mora-Poblete et al. (2023). The architecture of MLP was composed of (I) an input layer with n (number of molecular markers or spectral signatures) neurons, (II) three dense hidden layers, and (III) an output layer (dense layer for prediction). The neurons in the network are fully connected and perform non-linear transformations on the original input attributes. Additionally, the strength of the connection weights determines the contribution of each neuron to the overall network output. Deep learning models (CNN and MLP) were carried out in Python v3.11.6, Tensorflow v2.13.0, and Keras v3.0.0, considering the following hyperparameters according to Mora-Poblete et al. (2024): 200 epochs, CNN or MLP layers plus 3 dense hidden layers and 1 dropout layer (with 20% dropout), and rectified linear activation unit (ReLU) as the activation function method for training the models. Pseudocodes for implementing deep learning algorithms are provided in the methodology section of the **Supplementary Material**. In this study, we utilized a mid-level computing cluster equipped with 28 cores and 62 GB of RAM per core. While this setup represents a significant computational resource, it is increasingly feasible for many institutions through affordable cloud computing services, which have seen a reduction in costs in recent years (Yanamala, 2024). Moreover, all software used in this study is freely available, making it accessible to researchers irrespective of their financial constraints. It is important to note that although training deep learning models is resource-intensive, once the models are trained, they do not require ongoing computational resources for application. This allows for their deployment across various breeding programs without the need for additional training or adjustments, thus mitigating some of the initial computational demands.

#### 2.5.2 Cross-validation

The performance of Deep Learning models (CNN and MLP) using Uni/Multi-trait and Multi-omic approaches for predicting traits of industrial interest was assessed using 50 cycles of cross-validation. In each cycle, independent and non-overlapping groups for training (80%) and testing (20%) were randomly selected, ensuring that the data used for training were entirely separate from those used for testing. Furthermore, the random selection process in each cycle ensured that the training and testing sets remained independent across all cycles. The DL models were assessed to predict quantitative traits (Oil yield, TH, DBH, SC, and VOL), and categorical traits (BQ and ST). Prediction accuracy (PA) was assessed by calculating the mean of the Pearson correlation coefficient between observed and predicted traits. DL models (CNN or MLP) and types of omic datasets (SNPs, Haplotypes, NIR spectral absorbance, SNPs+NIR spectral absorbance, SNPs+Haplotypes, Haplotypes+NIR spectral absorbance, SNPs+Haplotypes+NIR spectral absorbance) were compared across Uni/Multi-trait and Uni/Multi-omic approaches. Significant differences in PA values between the omic dataset for Uni-trait and Multi-trait approaches were assessed by a general linear model (GLM) with Tukey’s *post hoc* multiple comparison tests (p<0.05). Significant differences in PA values of each procedure were assessed using the t-Student test (p<0.05, p<0.01, and p<0.001). Significant differences in PA values of the CNN model compared to the MLP model for the same assessed omic dataset and the same approach, were evaluated by the t-Student test. Statistical comparison tests were conducted using R 4.3.2 software (Core Development Team, 2020).

## 3 Results

### 3.1 Phenomic and genomic data

**Figure 1:**
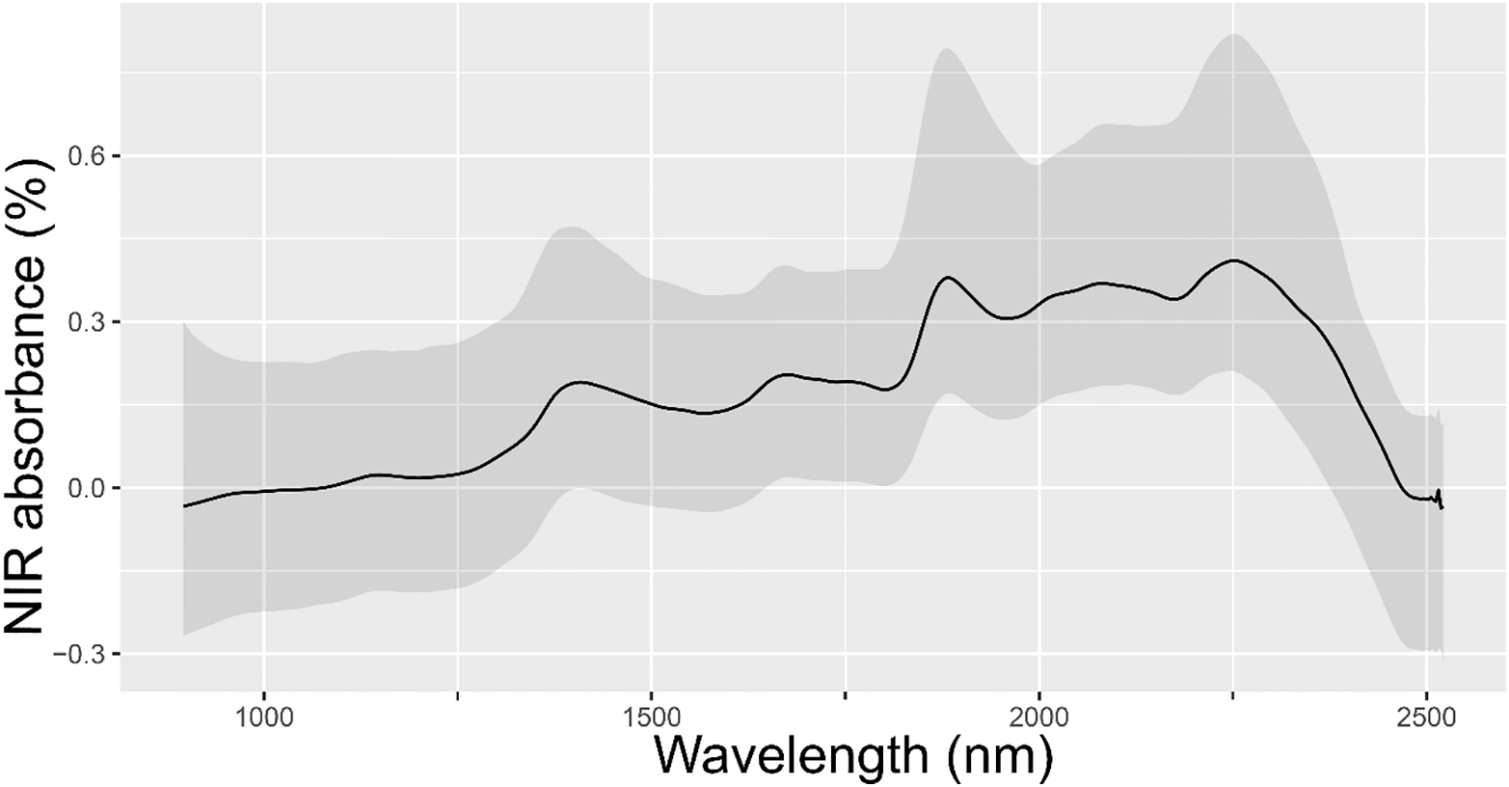
Mean of spectral absorbance values from the leaves of 339 randomly selected *E. globulus* trees from the study population. The mean and 95% confidence interval NIR spectral absorbance for all samples are colored in black and gray.

**Figure 1** shows the omic data related to NIR spectral absorbance from the leaves of randomly selected *E. globulus* individuals within the study population. Our results revealed that the spectral signature of leaves from this population exhibited four main peaks: between 1300-1500 nm, 1650-1800 nm, 1850-2000 nm, and 2200-2400 nm. On the other hand, the sample genotyping quality filters resulted in 14,442 high-quality SNPs for the individuals.

### 3.2 Foliar essential oil content, growth, and stem quality

Significant variations in foliar essential oil content and wood production-related traits were observed among the individuals. The essential oil content (oil yield) expressed as a percentage of mL of essential oil per g of leaf fresh weight (% v/fw) exhibited a range of 0.01-1.69 ± 0.001% v/fw (**Supplementary Table S2**), and the preliminary analysis of the main terpenes showed 8 major compounds, including 1,8-cineole, 1H-Cycloprop[e]azulene, α-Pinene, Globulol, α-Terpineol acetate, D-Limonene, Alloaromadendrene, and α-Gurjunene (**Supplementary Figure S1**). The quantitative traits related to wood production exhibited a range of variations, with values ranging from 3.6 to 18.0 m for TH, 4.3 to 22.7 cm for DBH, 0.60 to 1.77 m3 for VOL, and an index of 0.01 to 0.22 for SC (**Supplementary Table S2**).

**Figure 2:**
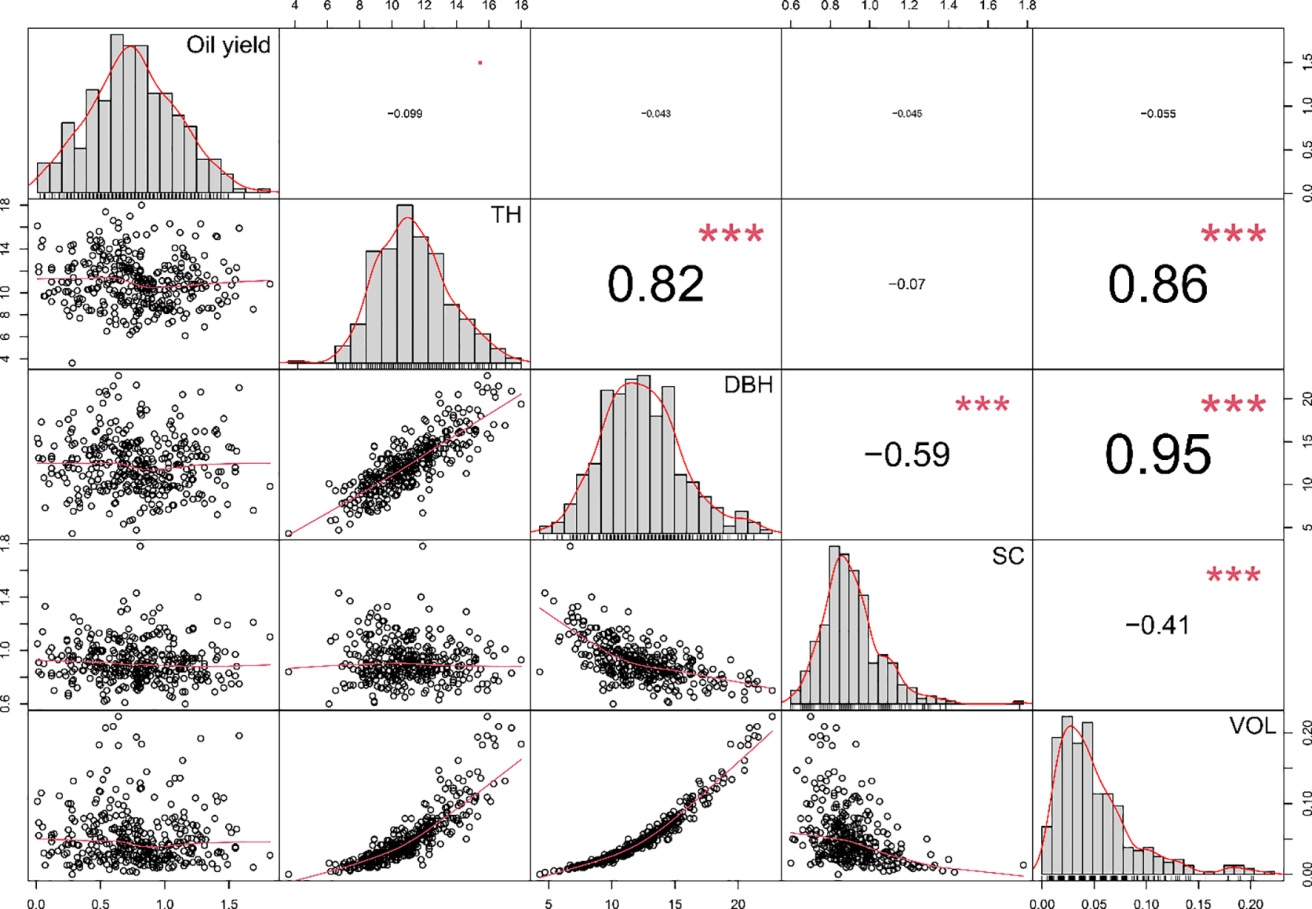
Pearson correlation coefficient between quantitative phenotypic traits of industrial interest assessed in the breeding population of *E. globulus* studied. The diagonal of the plot shows histograms and distributions of the observed phenotype values, while the lower off-diagonal displays scatter plots between the traits. Oil yield: essential oil content expressed as a percentage of mL of essential oil per g of leaf fresh weight (% v/fw); TH, tree height; DBH, diameter at breast height; SC, slenderness coefficient; VOL, stem volume. Significance levels of the correlation coefficients are indicated by *** for *p<*0.001.

The correlation analysis among quantitative traits indicated that essential oil content showed no significant correlation with any of the traits associated with wood production (**Figure 2**). This suggests that selection solely based on growth-related traits did not influence the essential oil content. Within the quantitative traits related to wood production, TH had a significant positive correlation with DBH (r=0.82) and VOL (r=0.86). Similarly, a significant positive correlation was observed between DBH and VOL (r=0.95). This coherence is expected, as the volume of a tree is inherently tied to its size, and DBH serves as a crucial measure of tree dimensions. A significant negative correlation was observed between SC and both DBH (r=-0.59) and VOL (r=-0.41), suggesting that with an increase in DBH (indicating greater thickness in relation to height), SC tends to decrease. Conversely, the categorical traits assessed (ST and BQ) exhibited a positive correlation (r=0.30), implying that, overall, trees with straighter stems tend to have branches of higher quality.

### 3.3 Genomic and pedigree-based heritability of phenotypic traits

**Table 1** presents heritability estimates for the essential oil content, stem quality, and growth-related traits in *E. globulus* trees, based on SNP markers and pedigree information. In this study, we found that genomic heritability values, as determined by SNPs, generally exceeded pedigree-based heritability. Genomic heritability ranged from 0.25 (for DBH and oil yield with Bayesian Ridge Regression) to as high as 0.71 (for DBH and ST with Bayes B), while pedigree-based heritability varied from 0.05 (for SC) to 0.88 (for BQ). Notably, oil yield was a moderately heritable trait, with genomic values spanning from 0.25 to 0.60, alongside a pedigree-based heritability estimate of 0.48. These findings underscore the substantial genetic variation present within the progeny trial for oil production.

**Table 1:** Estimates of heritability based on SNP markers ( $h_{g}^{2}$ ) and pedigree information ( $h_{a}^{2}$ ) for essential oil content, stem quality, and growth-related traits evaluated in 9-year-old *E. globulus* trees randomly selected from the study breeding population.

Trait	Genomic												Pedigree
	*Bayes A*			*Bayes B*			*Bayes C*			*BRR*			
	$h_{g}^{2}$	$\sigma_{g}^{2}$	$\sigma_{e}^{2}$	$h_{g}^{2}$	$\sigma_{g}^{2}$	$\sigma_{e}^{2}$	$h_{g}^{2}$	$\sigma_{g}^{2}$	$\sigma_{e}^{2}$	$h_{g}^{2}$	$\sigma_{g}^{2}$	$\sigma_{e}^{2}$	$h_{a}^{2}$
**EO**	0.34	0.05	0.10	0.60	0.16	0.11	0.56	0.14	0.11	0.25	0.03	0.10	0.48
**TH**	0.37	2.45	4.11	0.51	4.65	4.52	0.41	3.28	4.71	0.26	1.28	3.71	0.06
**DBH**	0.38	5.17	8.26	0.71	22.24	9.17	0.49	9.00	9.31	0.25	2.87	8.51	0.06
**VOL**	0.33	<0.01	<0.01	0.50	<0.01	<0.01	0.49	<0.01	<0.01	0.28	<0.01	<0.01	0.08
**ST**	0.36	0.57	1.00	0.71	2.43	1.00	0.58	1.39	1.00	0.28	0.39	1.00	0.61
**BQ**	0.46	0.84	1.00	0.67	2.02	1.00	0.54	1.15	1.00	0.35	0.55	1.00	0.88
**SC**	0.41	0.01	0.02	0.43	0.02	0.02	0.46	0.02	0.02	0.29	0.01	0.02	0.05

BRR, Bayesian Ridge Regression; $\sigma_{g}^{2}$ , genomic variance component; $\sigma_{e}^{2}$ , residual variance component; EO, oil yield; TH, tree height; DBH, diameter at breast height, SC, slenderness coefficient; VOL, stem volume; BQ, branching quality; ST, stem straightness.

### 3.4 Prediction accuracy based on uni/multi-trait and uni/multi-omic deep learning model

**Table 2** shows the mean prediction accuracy estimates of the DL models (including CNN and MLP) for the quantitative traits under study (oil yield, TH, DBH, SC, and VOL), as well as categorical traits (BQ and ST), measured in *E. globulus* trees. The predictions were based upon different omics datasets (SNPs, haplotypes, and NIR spectral data) considering both Uni-trait and Multi-trait approaches. The Multi-trait approach consistently evidenced superior PA values for the majority of the analyzed traits. For instance, in the case of TH, the MLP model employing the Multi-trait approach with the “Haplotypes” dataset achieved the highest prediction accuracy (0.772), significantly outperforming the Uni-trait approach with the same omic dataset (0.588). Likewise, the MLP model employing a Multi-trait approach exhibited improved accuracy in predicting oil yield, achieving a PA value of 0.699 when utilizing the “SNPs+Haplotypes+NIR spectral absorbance” data. Additionally, the MLP model employing a Multi-trait approach exhibited improved accuracy in predicting SC, achieving a PA of 0.616 when utilizing the “Haplotypes+NIR spectral absorbance “ data. On the other hand, the CNN model with a multi-trait approach and complemented with the “NIR spectral absorbance” data achieved PA values of 0.745 and 0.757 for the prediction of DBH and stem volume, respectively. Similarly, for the prediction of ST, the CNN model with a Multi-trait approach achieved a PA of 0.764 using the “SNP” data. In contrast, the Uni-trait approach demonstrated superior accuracy exclusively for the BQ trait, with a PA of 0.86 using the CNN model and the “SNPs+Haplotypes+NIR spectral absorbance” data. Notably, this PA value was not significantly different from the PA value obtained with the Multi-trait approach using the same deep learning model and omic data set (0.84).

**Table 2:** Mean of prediction accuracy estimates for Uni/Multi-trait and Uni/Multi-omic deep learning models assessed to predict phenotypic traits of industrial interest in *E. globulus*.

		*Quantitative traits*										*Categorical traits*			
		Oil yield		TH		DBH		SC		VOL		BQ		ST	
	*Omic-data set*	*CNN*	*MLP*	*CNN*	*MLP*	*CNN*	*MLP*	*CNN*	*MLP*	*CNN*	*MLP*	*CNN*	*MLP*	*CNN*	*MLP*
***Uni-trait***	*SNPs*	0.623 a,*	0.568 a	0.597 a	0.589 a	0.626 a	0.614 a	0.608 a	0.573 a	0.584 a	0.584 a	0.807 abc	0.807 a	0.726 a	0.709 a,*
	*Haplotypes*	0.592 a	0.570 a	0.608 a	0.588 a	0.599 a,*	0.564 a,*	0.577 a	0.573 a,*	0.560 a	0.603 a	0.785 bc	0.801 a	0.709 a	0.728 a
	*NIR spectral absorbance*	0.595 a	0.638 a	0.602 a	0.612 a,*	0.594 a,*	0.564 a,*	0.570 a	0.577 a	0.626 a,*	0.594 a	0.775 c	0.840 a,+	0.701 a	0.737 a
	*SNPs+NIR spectral absorbance*	0.565 a	0.596 a,*	0.596 a	0.611 a	0.598 a	0.608 a	0.556 a	0.596 a	0.613 a	0.578 a	0.850 ab	0.854 a	0.751 a	0.721 a
	*SNPs+Haplotypes*	0.637 a	0.629 a	0.576 a	0.616 a,*	0.579 a	0.622 a	0.605 a	0.579 a	0.632 a,*	0.609 a	0.793 abc	0.791 a	0.749 a	0.747 a
	*Haplotypes+NIR spectral absorbance*	0.600 a	0.601 a	0.624 a,+,*	0.548 a	0.590 a	0.585 a	0.563 a	0.597 a	0.569 a	0.597 a	0.824 abc	0.812 a	0.703 a	0.756 a,+
	*SNPs+Haplotypes+NIR spectral absorbance*	0.657 a,*	0.637 a	0.623 a	0.601 a	0.586 a,*	0.566 a,*	0.594 a	0.574 a	0.606 a	0.588 a	**0.861 a**	0.838 a	0.749 a	0.728 a
***Multi-trait***	*SNPs*	0.563 bc	0.572 d	0.605 bc	0.612 b	0.552 c	0.600 bc,+	0.599 a	0.598 ab	0.576 c	0.590 abc	0.793 a	0.785 a	**0.764 a**	0.754 a
	*Haplotypes*	0.604 ab,+	0.540 d	0.632 b	0.772 a,+,*	0.539 c	0.657 a,+	0.558 a	0.530 b	0.538 c	0.619 ab,+	0.812 a	0.803 a	0.721 ab	0.703 a
	*NIR spectral absorbance*	0.604 ab	0.673 ab	0.718 a,+,*	0.548 cd	**0.745 a,+**	0.645 ab	0.572 a	0.568 ab	**0.757 a,+**	0.650 a	0.803 a	0.785 a	0.741 ab	0.724 a
	*SNPs+NIR spectral absorbance*	0.624 ab,+	0.538 d	0.587 bc	0.553 bcd	0.630 b,+	0.572 cd	0.541 a	0.599 ab,+	0.638 b,+	0.536 c	0.816 a	0.825 a	0.707 ab	0.713 a
	*SNPs+Haplotypes*	0.668 a,+	0.622 c	0.559 bc	0.535 d	0.545 c	0.562 cd	0.593 a	0.551 ab	0.553 c	0.570 bc	0.793 a	0.801 a	0.722 ab	0.713 a
	*Haplotypes+NIR spectral absorbance*	0.582 bc	0.633 bc,+	0.543 c	0.563 bcd	0.564 c	0.580 cd	0.549 a	**0.616 a,+**	0.549 c	0.571 bc	0.819 a	0.826 a	0.693 b	0.729 a
	*SNPs+Haplotypes+NIR spectral absorbance*	0.525 c	**0.699 a,+**	0.605 bc	0.604 bc	0.528 c	0.531 d	0.554 a	0.570 ab	0.640 b	0.604 ab	0.841 a	0.837 a	0.734 ab	0.719 a

PA, prediction accuracy; Oil yield, essential oil content expressed as a percentage of mL of essential oil per g of leaf fresh weight (% v/fw); TH, tree height; DBH, diameter at breast height; SC, slenderness coefficient; VOL, stem volume; BQ, branching quality; ST, stem straightness. Different letters (a, b, c, d) show significant differences in PA values between the omic-data set for Uni-trait and Multi-trait approaches, according to Tukey’s test (p<0.05). + (p<0.05) show significant differences in PA values of the CNN model compared to the MLP model for the same assessed omic-data set and the same trait approach, according to the t-Student test. *(p<0.05) show significant differences in PA values of the assessed omic-data set of the Uni-trait approach compared to the Multi-trait approach, according to the t-Student test. The best PA values for each trait evaluated are highlighted in bold.

The results of this study revealed that Multi-trait models, which combine SNPs, haplotypes, NIR spectral absorbance, or the combination of both omics data, consistently outperformed the Uni-trait approach in six out of seven traits (oil yield, TH, DBH, SC, VOL, and ST). In contrast, individual omics databases (not combined with other data) attained higher PA for the Multi-trait approach in four out of seven traits (TH, DBH, VOL, and ST). NIR spectral absorbance data, either alone or combined with other omics data, resulted in the highest PA estimates for a substantial majority of traits (71% of traits for Multi-trait and 57% of traits for Uni-trait). Furthermore, NIR spectral absorbance data were the best selection for the CNN or MLP models with a Multi-trait approach since in most of the traits evaluated (except for TH) no significant differences were observed between this data and those omics data or combinations that presented the best PA values. Interestingly, the “SNPs+Haplotypes+NIR spectral absorbance” dataset exhibited statistically significant differences from all other omics datasets, except NIR spectral absorbance alone. This suggests that both NIR spectral absorbance alone or in combination with other omics (SNP and haplotype datasets) may be valuable for enhancing essential oil content prediction within the *Eucalyptus* genus.

The statistical analysis revealed significant differences between the PA values of CNN and MLP models in multiple instances. These findings strongly suggest that the selection of the deep learning model can have a substantial impact on prediction accuracy, contingent upon both the omic data set and the approach utilized. This highlights the importance of considering the specific traits of *Eucalyptus* species when selecting the most appropriate model.

## 4 Discussion

While our study successfully applied deep learning models to predict traits of industrial interest, it did not delve into the identification of genetic variants associated with specific phenotypic traits. Instead, we focused on leveraging genomic selection to enhance the accuracy of predicting complex traits based on genomic and phenomic data. This approach is instrumental in breeding programs as it facilitates the early identification of superior individuals by predicting desirable phenotypic traits. By improving the precision of these predictions, we can accelerate the breeding process, enhance selection accuracy, and manage large populations more efficiently. Additionally, it aids in better managing genetic diversity, integrating multiple traits, simulating various breeding scenarios, and predicting trait evolution within the population (Grattapaglia, 2017).

In practical breeding programs, our predictions can be utilized to select traits such as essential oil content, wood quality, and adaptability to climate change. For instance, accurate predictions of branching quality and growth traits can guide the selection of individuals who will likely produce higher-quality wood or more resilient trees. This is particularly relevant in addressing challenges such as the demand for high-quality wood and essential oils, as well as ensuring sustainability in forest production. Our findings are consistent with previous research highlighting the role of genomic selection in advancing the genetic enhancement of *Eucalyptus* and other species (Myburg et al., 2014; Ballesta et al., 2018, Ballesta et al., 2019, Ballesta et al., 2022; Mora-Poblete et al., 2021; Mora-Poblete et al., 2024).

### 4.1 Predicting traits in *Eucalyptus* using NIR spectral data

In *E. globulus* and other *Eucalyptus* species of forestry interest, phenomic tools such as NIR spectroscopy have been employed to predict wood chemical properties, including lignin content (total, insoluble, and soluble), syringyl-guaiacyl ratio, and the content of different monosaccharides. These predictions contribute significantly to the classification of species, families, and clones, as highlighted by Hodge et al. (2018). Furthermore, it has been described that different leaf spectral reflectance indexes that include NIR data have provided information on several physiological traits of agronomic interest traits in *Eucalyptus* and other species (Lobos and Poblete-Echeverría, 2017; Ballesta et al., 2022). Our findings indicated that the NIR spectra were similar to previous reports illustrating a distinctive spectral signature of *E. globulus* leaves characterized by four main peaks: between 1300-1500 nm, 1650-1800 nm, 1850-2000 nm, and 2200-2400 nm (Wilson et al., 2001; Castillo et al., 2008). Furthermore, NIR spectral peaks within the ranges of 1650-1800 nm and 2200-2400 nm have been reported to be associated with essential oil and 1,8-cineole content (Wilson et al., 2001). Similarly, the association between leaf NIR spectral data and foliar essential oil content has been previously utilized to differentiate *E. globulus*, *E. nitens*, and their hybrid F1 (Humphreys et al., 2008). Our study considered DL models that used the full foliar NIR spectra (900-2500 nm) as phenomic data for the prediction of traits associated with wood and essential oil content. Recently, Ballesta et al. (2022) proposed the use of the full foliar NIR spectral data to improve genomic prediction of other secondary metabolites such as cyanogenic glycosides content in *E. cladocalyx*. Moreover, in individuals of *E. cladocalyx*, it has been emphasized that the use of foliar NIR spectral data together with DL models improves the ability to discriminate and assign individuals to specific subpopulations (genetic structure), facilitating the implementation and application of population structure studies on a large scale (Maldonado et al., 2022).

### 4.2 Essential oil content variation in breeding population of *E. globulus*

Understanding the genetic basis of essential oil production in *E. globulus* is crucial for optimizing breeding strategies in this economically important species. In this study, the observed difference between genomic and pedigree-based heritability highlights the importance of leveraging genomic data to accurately quantify genetic contributions to complex traits, an issue emphasized by Ballesta et al. (2020). The observed moderate-to-high heritability of essential oil content suggests that genetic factors play a substantial role in determining essential oil yields in *E. globulus*. This substantial genetic variation within the breeding population underscores the importance of further exploration into the underlying genetic mechanisms and environmental factors influencing this trait. The observed variation in essential oil content is consistent with previous findings for *E. globulus*, with studies reporting yield ranges of 0.80-2.10% v/fw in Ethiopia (Subramanian et al., 2012; Kassahun and Feleke, 2019), 1.31 ± 0.14% v/fw in Argentina (Russo et al., 2015), 2.12% v/fw in Morocco (Zrira et al., 1992), 1.70-2.20% v/fw in Portugal (Silvestre et al., 1997), 2.20% v/fw in Vietnam (Ngo et al., 2020), and 2.50% v/fw in Algeria (Daroui-Mokaddem et al., 2010). The difference in essential oil content (% v/fw) between *E. globulus* individuals from different regions of the world is because this trait depends upon variations in complex quantitative traits such as foliar oil concentration, foliar biomass, and environmental adaptability (Kainer et al., 2015). This is consistent with our correlation results between quantitative traits, which indicated that essential oil content has no significant correlation with stem quality or growth-related traits.

### 4.3 Improving the prediction accuracy of foliar essential oil content, growth, and stem quality in *E. globulus* using multi-trait deep learning models

The results showed that the PA values depended on the DL models (CNN and MLP), the type of approach (Uni-trait and Multi-trait), and the omic data set (SNPs, haplotypes, NIR spectral absorption, or the combination of these omic data). Previous studies have shown that different DL model architectures could have a significant impact on prediction accuracy in *Eucalyptus* (Maldonado et al., 2020). Therefore, it is important to take these considerations into account when implementing DL models for phenotypic trait prediction in *E. globulus*. Our results demonstrate that DL models that integrate phenotypic traits in Multi-trait approach increase prediction accuracy compared to Uni-trait approach. In this sense, 86% of traits showed the highest PA values in the Multi-trait approach. Among these, 83% had significantly greater efficiencies compared to their Uni-train counterparts. Several studies have reported that the Multi-trait approach generally offers better prediction accuracy compared to the Uni-trait approach, particularly when the evaluated traits are correlated (Sandhu et al., 2022; Mora-Poblete et al., 2023). This observation aligns with our results, as the highest PA values for the Multi-trait approach were associated with quantitative traits that exhibited positive correlations, such as TH, DBH, SC, and VOL (**Figure 2**). In contrast, the Uni-trait approach demonstrated high PA values for traits with low or no correlation with other evaluated traits, such as oil yield, BQ, and ST. Notably, BQ showed the highest PA value using the Uni-trait approach, though there were no significant differences compared to the PA value obtained with the Multi-trait approach using the same deep learning model and omics data set.

Branching quality exhibited higher heritability across most models used, including Bayes A and Bayesian Ridge Regression (BRR) based on genomic data, as well as models based on pedigree. As expected, traits with high heritability, such as branching quality, show greater prediction accuracies compared to traits with lower heritability. This pattern is supported by similar findings in the literature, which consistently demonstrate a strong relationship between prediction accuracy and trait heritability (Kaler et al., 2022; Cui et al., 2020). Our results underscore the importance of considering heritability when evaluating the precision of predictive models, highlighting the benefits of a Multi-trait approach for traits with positive correlations and the utility of the Uni-trait approach for less correlated traits. These findings reinforce previous research that has shown the advantages of evaluating multiple phenotypic traits simultaneously for predicting complex traits in plants, including *Eucalyptus* (Maldonado et al., 2020; Mora-Poblete et al., 2023).

To the best of our knowledge, this work represents a pioneering effort in employing DL models to improve the prediction accuracy of traits associated with essential oil content in the genus *Eucalyptus*. Previously, Kainer et al. (2018) assessed the predictive accuracy of genomic models of the foliar terpene traits, including total leaf oil concentration in *E. polybractea* employing different methodologies such as traditional pedigree-based Additive Best Linear Unbiased Prediction (ABLUP), Genomic BLUP (GBLUP), Bayes B genomic prediction model, and a form of GBLUP based on weighting markers according to their influence on traits (BLUP|GA). Their findings indicated that the predictive performance varied across different terpene traits. Interestingly, they reported that the predictive ability was higher with Bayes B and BLUP|GA for individual terpene traits, such as α-pinene and 1,8-cineole concentration, with values of 0.59 and 0.73, respectively. The Bayes B method assumes that each marker has its own variance, and the phenotypic variance is explained by loci with effects of different magnitudes (Wang et al., 2018). For aggregate traits such as total leaf oil concentration, the study of Kainer et al. (2018) found that the predictive value was comparatively lower (0.38). Our results indicate that the MLP model with a Multi-trait approach and utilizing the combined “SNPs+Haplotypes+NIR spectral absorbance” dataset presented superior predictive values (0.699) for the essential oil content.

Although the comparison of prediction values may be biased due to the differing omics datasets used in Bayesian models from previous studies, our findings are consistent with those of Mora-Poblete et al. (2023), who observed that Multi-trait deep learning models surpassed Bayesian and GBLUP predictive models in both capturing genetic variation and prediction accuracy. Regarding growth-related traits and stem quality, our DL models (including CNN and MLP) exhibited higher PA values for traits such as ST, DBH, TH, and BQ compared to previous evaluations of the progeny trial. For instance, Ballesta et al. (2019) used Bayesian genomic models (BA, BB, BC, BL, BRR) incorporating the effects of haplotypes and SNPs to predict quantitative wood-related traits, reporting PA values of 0.580, 0.460, 0.440, and 0.330 for ST, DBH, TH, and BQ, respectively. Maldonado et al. (2020) employed DL techniques, specifically Long Short-Term Memory Network (LSTM) and Bayesian Regularized Neural Network (BRNN) models, focusing solely on the effects of SNPs in *E. globulus*. Their study revealed that the DL model, particularly the LSTM variant, achieved the highest PA values (0.460 to 0.557), demonstrating the superior performance of DL methods in predicting wood-related traits in *E. globulus* compared to traditional approaches. This underscores the potential of DL techniques in enhancing the accuracy of genetic prediction models for complex quantitative traits, thereby facilitating more efficient breeding strategies in forestry applications. Deep learning has become a powerful tool across various scientific domains, offering innovative approaches to tackle complex problems. For instance, it has been utilized to enhance the prediction of industrial yield phenotypes in trees (Maldonado et al., 2020), classify proteins based on sequence data (Kha et al., 2022), and develop diagnostic and treatment strategies for cancer patients (Tran et al., 2024). Deep learning models, as end-to-end systems, are capable of processing high-dimensional raw input data, enabling superior feature extraction and learning capabilities compared to traditional methods (Tran et al., 2024). This allows deep learning models to excel in handling high-dimensional omics data, producing more robust and accurate predictive results than conventional machine learning approaches. Our findings corroborate the superiority of Multi-trait models in terms of prediction accuracy, as demonstrated even for uncorrelated traits (Mora-Poblete et al., 2023). These results could be attributed to the ability of deep learning to capture intricate interactions within its hidden layers, eliminating the need for explicit covariate specification (Montesinos-López et al., 2018; Mora-Poblete et al., 2023). Interestingly, NIR spectral data consistently yielded the highest average prediction accuracy across traits in the Multi-trait approach. This finding is consistent with other studies in which NIR spectral absorbance increased the accuracy of predicting various traits in *Eucalyptus* (Mora-Poblete et al., 2021; Ballesta et al., 2022; Mora-Poblete et al., 2024). Additionally, this result aligns with Rincent et al. (2018), who employed NIR reflectance as a method to indirectly capture endophenotypic variants and compute relationship matrices for predicting complex traits in breeding populations, demonstrating its effectiveness in prediction models.

The success of deep learning in predicting *Eucalyptus* traits suggests its potential applicability to other forest tree species. Additionally, it highlights the potential of NIR spectral information as a low-cost phenotyping tool (Rincent et al., 2018) that enables the acquisition of omics data on a large scale and improves the prediction accuracy of various traits of industrial interest. Furthermore, this study represents a pioneering effort in experimentally testing deep learning models trained on multi-omics datasets that combine genomic information (SNPs and Haplotypes) with NIR spectral absorbance for phenotypic trait prediction. We propose this innovative approach as a valuable complement to traditional methods of genomic and phenomic prediction.

## 5 Conclusion

Accurate prediction of industrial traits in *Eucalyptus* species is crucial for selecting desirable genotypes and advancing genetic improvement. Our results demonstrate that Deep Learning models (CNN and MLP), incorporating a Multi-trait approach and NIR spectral absorbance, can significantly improve prediction accuracy within tree breeding programs. This has the potential not only to facilitate the production of genetically improved seeds and individuals of *E. globulus* with enhanced growth traits and stem quality but also to improve the traits related to essential oil content, a key non-timber forest product. This, in turn, promotes sustainable production and consumption across various industrial applications. The insights and findings from this research significantly contribute to understanding omics-assisted deep learning models as a breeding strategy to improve traits of industrial interest in *Eucalyptus globulus*, such as wood and essential oil production. These advancements not only foster progress in the field of plant science but also enable more efficient and targeted breeding efforts, ultimately driving innovation and sustainability in *Eucalyptus* plantations.

## Data Availability

The data presented in the study are deposited in the Figshare repository, https://figshare.com/articles/dataset/Supplementary_Omics_data_used_in_this_study_/27165612.
