# Supplementary material for: Enhancing prediction accuracy of foliar essential oil content, growth, and stem quality in Eucalyptus globulus using multi-trait deep learning models
Source: Front Plant Sci. 2024 Oct 10;15:1451784. doi: 10.3389/fpls.2024.1451784 (PMC11499176; doi:10.3389/fpls.2024.1451784)
Supplement: Supplementary file 1 [file DataSheet1.pdf]

Supplementary Material

Enhancing prediction accuracy of foliar essential oil content, growth, and stem quality in *Eucalyptus globulus* using multi-trait deep learning models

Daniel Mieres-Castro[1], Carlos Maldonado[2], Freddy Mora-Poblete[1*]

[1]Laboratory of Genomics and Forestry Biotechnology, Institute of Biological Sciences, University of Talca, Lircay Avenue S/N, 3461334 Talca, Chile

[2]Centro de Genómica y Bioinformática, Facultad de Ciencias, Universidad Mayor, Camino La Pirámide 5750, Huechuraba, 8580745, Santiago, Chile

Supplementary methodology. Pseudocode for Implementing Deep Learning Algorithms.

```
X1_valid, X1_train, Y1_valid, Y1_train = train_test_split(X_temp, Y1_temp, test_size=0.80)

########UNI-TRAIT

####categorical traits

#MLP

model_mlp = Sequential()

model_mlp.add(Dense(units=6252, activation="relu", input_dim=X1_train.shape[1]))

model_mlp.add(Dense(units=3072, activation='relu'))

model_mlp.add(Dense(units=1280, activation='relu'))

model_mlp.add(Dense(units=Y1_train.shape[1], activation='softmax'))

model_mlp.compile(loss='categorical_crossentropy', optimizer='adam',metrics=['accuracy'])

model_mlp.fit(X1_train, Y1_train, epochs=200, verbose=0)

model_mlp.evaluate(X1_valid, Y1_valid)


#CNN

model_cnn = Sequential()

model_cnn.add(Conv1D(filters=2048, kernel_size=3, strides=3, input_shape=(X1_train.shape[1],1)))

model_cnn.add(Conv1D(1024, kernel_size=3, activation="relu"))

model_cnn.add(Dropout(0.2))

model_cnn.add(MaxPooling1D(pool_size=2))

model_cnn.add(Flatten())

model_cnn.add(Dense(512))

model_cnn.add(Activation("relu"))

model_cnn.add(Dense(128))

model_cnn.add(Activation('relu'))

model_cnn.add(Dense(units=Y1_train.shape[1], activation='softmax'))

model_cnn.compile(loss='categorical_crossentropy', optimizer='adam',metrics=['accuracy'])

model_cnn.fit(X1_train, Y1_train, epochs=200, verbose=0)

model_cnn.evaluate(X1_valid, Y1_valid)


####quantitative traits

#MLP

model_mlp = Sequential()

model_mlp.add(Dense(units=6252, activation="relu", input_dim=X1_train.shape[1]))
```

```
model_mlp.add(Dense(units=3072, activation='relu'))
model_mlp.add(Dense(units=1280, activation='relu'))
model_mlp.add(Dense(units=1, activation='linear'))
model_mlp.compile(loss='mse', optimizer="adam")
model_mlp.fit(X1_train, Y1_train, epochs=200, verbose=0)
model_mlp.predict(X1_valid)

#CNN
model_cnn = Sequential()
model_cnn.add(Conv1D(filters=2048, kernel_size=3, strides=3, input_shape=(X1_train.shape[1],1)))
model_cnn.add(Conv1D(1024, kernel_size=3, activation="relu"))
model_cnn.add(Dropout(0.2))
model_cnn.add(MaxPooling1D(pool_size=2))
model_cnn.add(Flatten())
model_cnn.add(Dense(512))
model_cnn.add(Activation("relu"))
model_cnn.add(Dense(128))
model_cnn.add(Activation('linear'))
model_cnn.add(Dense(1))
model_cnn.compile(loss='mse', optimizer="adam")
model_cnn.fit(X1_train, Y1_train, epochs=200, verbose=0)
model_cnn.predict(X1_valid)
```

########MULTI-TRAIT

```
####categorical traits
#MLP
input_layer = Input(shape=(X1_train.shape[1],))
hidden_layer_1 = Dense(6252, activation='relu')(input_layer)
hidden_layer_2 = Dense(3072, activation='relu')(hidden_layer_1)
hidden_layer_3 = Dense(1280, activation='relu')(hidden_layer_2)
output_layer_1 = Dense(5, activation='softmax', name='output_1')(hidden_layer_3)
output_layer_2 = Dense(6, activation='softmax', name='output_2')(hidden_layer_3)
output_layer_3 = Dense(2, activation='softmax', name='output_3')(hidden_layer_3)
output_layer_4 = Dense(4, activation='softmax', name='output_4')(hidden_layer_3)
model_mlp = Model(inputs=input_layer, outputs=[output_layer_1, output_layer_2, output_layer_3, output_layer_4])
```

```
model_mlp.compile(optimizer='adam',loss={'output_1': 'categorical_crossentropy', 'output_2': 'categorical_crossentropy', 'output_3': 'categorical_crossentropy', 'output_4': 'categorical_crossentropy'},metrics={'output_1': 'accuracy', 'output_2': 'accuracy', 'output_3': 'accuracy', 'output_4': 'accuracy'})

model_mlp.fit(X1_train, {'output_1': Y1_depredacion_train, 'output_2': Y1_RECT_train, 'output_3': Y1_FLECHA_train, 'output_4': Y1_CRAMA_train}, epochs=200)

model_mlp.evaluate(X1_valid, {'output_1': Y1_depredacion_valid, 'output_2': Y1_RECT_valid, 'output_3': Y1_FLECHA_valid, 'output_4': Y1_CRAMA_valid})


#CNN

input_layer = Input(shape=(X1_train.shape[1],1))

hidden_layer_1 = Conv1D(2048, kernel_size=3, activation="relu")(input_layer)

hidden_layer_2 = Conv1D(1024, kernel_size=3, activation="relu")(hidden_layer_1)

hidden_layer_2 = Dropout(0.2)(hidden_layer_2)

hidden_layer_3 = MaxPooling1D(pool_size=2)(hidden_layer_2)

flatten = Flatten()(hidden_layer_3)

hidden_layer_4 = Dense(512, activation='relu')(flatten)

hidden_layer_5 = Dense(128, activation='relu')(hidden_layer_4)

output_layer_1 = Dense(5, activation='softmax', name='output_1')(hidden_layer_4)

output_layer_2 = Dense(6, activation='softmax', name='output_2')(hidden_layer_4)

output_layer_3 = Dense(2, activation='softmax', name='output_3')(hidden_layer_4)

output_layer_4 = Dense(4, activation='softmax', name='output_4')(hidden_layer_4)

model_cnn = Model(inputs=input_layer, outputs=[output_layer_1, output_layer_2, output_layer_3, output_layer_4])

model_cnn.compile(optimizer='adam',loss={'output_1': 'categorical_crossentropy', 'output_2': 'categorical_crossentropy', 'output_3': 'categorical_crossentropy', 'output_4': 'categorical_crossentropy'},metrics={'output_1': 'accuracy', 'output_2': 'accuracy', 'output_3': 'accuracy', 'output_4': 'accuracy'})

model_cnn.fit(X1_train, {'output_1': Y1_depredacion_train, 'output_2': Y1_RECT_train, 'output_3': Y1_FLECHA_train, 'output_4': Y1_CRAMA_train}, epochs=200)

model_cnn.evaluate(X1_valid, {'output_1': Y1_depredacion_valid, 'output_2': Y1_RECT_valid, 'output_3': Y1_FLECHA_valid, 'output_4': Y1_CRAMA_valid})


####quantitative traits

#MLP

model_mlp = Sequential()

model_mlp.add(Dense(units=6252, activation="relu", input_dim=X1_train.shape[1]))

model_mlp.add(Dense(units=3072, activation='relu'))

model_mlp.add(Dense(units=1280, activation='relu'))

model_mlp.add(Dense(units=1, activation='linear'))

model_mlp.compile(loss='mse', optimizer="adam")
```

```
model_mlp.fit(X1_train, Y1_train, epochs=200, verbose=0)
model_mlp.predict(X1_valid)

#CNN
model_cnn = Sequential()
model_cnn.add(Conv1D(filters=2048, kernel_size=3, strides=3, input_shape=(X1_train.shape[1],1)))
model_cnn.add(Conv1D(1024, kernel_size=3, activation="relu"))
model_cnn.add(Dropout(0.2))
model_cnn.add(MaxPooling1D(pool_size=2))
model_cnn.add(Flatten())
model_cnn.add(Dense(512))
model_cnn.add(Activation("relu"))
model_cnn.add(Dense(128))
model_cnn.add(Activation('linear'))
model_cnn.add(Dense(1))
model_cnn.compile(loss='mse', optimizer="adam")
model_cnn.fit(X1_train, Y1_train, epochs=200, verbose=0)
model_cnn.predict(X1_valid)
```

Supplementary Table 1. Summary of foliar essential oil content and wood production in 339 randomly selected *Eucalyptus globulus* individuals from the breeding population.

Sample ID	***Quantitative traits***					***Categorical traits***	
	**Oil yield (% v/fw)**	**TH (m)**	**DBH (cm)**	**VOL (m)**	**SC (TH/DBH)**	**ST**	**BQ**
**B1C1**	0.50 ± 0.02	8.40	11.30	0.743	0.03	1	1
**B1C2**	0.93 ± 0.01	8.80	10.00	0.880	0.02	3	1
**B1S1**	0.32 ± 0.01	9.40	13.00	0.723	0.04	3	1
**B1S15**	0.78 ± 0.01	6.70	4.70	1.426	0.00	3	4
**B1S18**	0.24 ± 0.02	10.90	10.20	1.069	0.03	1	1
**B1S19**	0.62 ± 0.01	7.60	8.80	0.864	0.01	4	1
**B1S20**	0.80 ± 0.00	9.70	12.80	0.758	0.04	3	3
**B1S21**	1.06 ± 0.01	10.80	16.50	0.655	0.08	4	4
**B1S22**	0.55 ± 0.00	10.70	12.30	0.870	0.04	3	3
**B1S24**	0.85 ± 0.02	10.20	12.20	0.836	0.04	4	4
**B1S27**	0.86 ± 0.00	10.00	13.00	0.769	0.04	3	1
**B1S28**	0.65 ± 0.02	12.50	11.50	1.087	0.04	4	3
**B1S29**	0.57 ± 0.01	10.30	12.60	0.817	0.04	4	1
**B1S30**	0.40 ± 0.02	10.30	8.80	1.170	0.02	1	1
**B1S31**	0.98 ± 0.02	11.10	11.40	0.974	0.04	3	1
**B1S33**	0.98 ± 0.00	8.80	10.70	0.822	0.03	3	2
**B1S35**	1.22 ± 0.02	13.00	14.20	0.915	0.07	3	2
**B1S36**	1.49 ± 0.01	9.20	7.70	1.195	0.01	3	1

**B1S41**	0.79 ± 0.02	11.50	12.80	0.898	0.05	3	2
**B1S42**	0.69 ± 0.00	8.90	11.00	0.809	0.03	1	3
**B1S43**	0.23 ± 0.00	10.10	12.50	0.808	0.04	4	3
**B1S44**	0.55 ± 0.01	12.90	20.50	0.629	0.15	4	2
**B1S51**	0.49 ± 0.02	13.80	17.80	0.775	0.12	4	2
**B1S52**	0.64 ± 0.01	16.40	20.20	0.812	0.18	3	4
**B1S55**	1.01 ± 0.01	9.80	13.60	0.721	0.05	3	2
**B1S58**	0.61 ± 0.01	10.20	10.40	0.981	0.03	3	1
**B1S64**	1.16 ± 0.01	6.10	10.10	0.604	0.02	3	1
**B1S65**	0.18 ± 0.01	14.00	17.10	0.819	0.11	3	4
**B1S66**	0.86 ± 0.01	10.40	11.00	0.945	0.03	1	1
**B1S7**	0.57 ± 0.02	14.00	14.90	0.940	0.08	3	2
**B1SD10**	1.29 ± 0.01	8.60	8.80	0.977	0.02	4	3
**B1SD13**	1.17 ± 0.01	13.30	14.20	0.937	0.07	3	3
**B1SD14**	0.39 ± 0.00	8.70	9.50	0.916	0.02	1	1
**B1SD25**	0.87 ± 0.00	9.50	9.50	1.000	0.02	2	3
**B1SD38**	0.64 ± 0.00	15.90	22.70	0.700	0.22	4	2
**B1SD40**	1.52 ± 0.02	10.70	14.30	0.748	0.06	4	3
**B1SD5**	0.77 ± 0.02	13.60	12.50	1.088	0.06	1	4
**B1SD6**	0.81 ± 0.02	11.90	6.70	1.776	0.01	3	4
**B1SD9**	1.11 ± 0.02	10.60	12.30	0.862	0.04	2	2
**B2C1**	1.04 ± 0.02	8.80	13.30	0.662	0.04	3	1
**B2C4a**	1.06 ± 0.02	7.40	7.60	0.974	0.01	1	1

**B2C4b**	1.55 ± 0.01	9.70	12.00	0.808	0.04	4	4
**B2S15**	0.36 ± 0.01	9.4	11	0.855	0.03	1	1
**B2S16**	0.90 ± 0.02	11.10	13.00	0.854	0.05	3	1
**B2S18**	1.12 ± 0.00	11.50	12.30	0.935	0.05	3	4
**B2S21**	0.78 ± 0.00	12.50	15.00	0.833	0.08	3	3
**B2S22**	1.26 ± 0.00	11.10	13.80	0.80	0.06	3	4
**B2S24**	0.61 ± 0.01	8.10	9.80	0.827	0.02	3	1
**B2S29**	1.20 ± 0.01	9.50	11.10	0.856	0.03	4	2
**B2S3**	0.45 ± 0.01	12.20	14.40	0.847	0.07	1	3
**B2S30**	0.96 ± 0.01	10.30	8.80	1.170	0.02	1	1
**B2S31**	1.41 ± 0.00	15.10	15.90	0.950	0.10	4	4
**B2S34**	0.81 ± 0.01	8.40	10.10	0.832	0.02	4	3
**B2S35**	1.22 ± 0.01	10.70	11.40	0.939	0.04	5	4
**B2S36**	0.62 ± 0.02	14.60	17.40	0.839	0.12	5	2
**B2S37**	0.53 ± 0.00	8.20	10.00	0.820	0.02	4	3
**B2S41**	0.72 ± 0.01	12.00	14.40	0.833	0.07	3	1
**B2S43**	1.12 ± 0.00	11.70	13.50	0.867	0.06	4	3
**B2S44b**	0.27 ± 0.02	14.30	16.10	0.888	0.10	4	3
**B2S52**	1.13 ± 0.01	12.30	13.90	0.885	0.06	4	3
**B2S54**	0.52 ± 0.00	13.00	19.50	0.667	0.13	1	2
**B2S55**	1.21 ± 0.00	10.10	12.00	0.842	0.04	4	1
**B2S56**	0.69 ± 0.00	8.90	14.50	0.614	0.05	3	1

**B2S57**	0.71 ± 0.01	9.10	10.00	0.910	0.02	4	1
**B2S63**	1.34 ± 0.00	8.60	11.10	0.775	0.03	1	1
**B2S64**	0.82 ± 0.02	18.00	19.40	0.928	0.18	1	3
**B2S65**	1.01 ± 0.02	8.40	9.90	0.848	0.02	4	3
**B2S66**	0.90 ± 0.00	9.50	10.50	0.905	0.03	4	4
**B2SD12**	1.31 ± 0.00	10.80	12.00	0.900	0.04	4	4
**B2SD13**	1.56 ± 0.00	12.30	13.90	0.885	0.06	3	3
**B2SD14**	1.69 ± 0.00	8.50	7.00	1.214	0.01	1	3
**B2SD6**	0.65 ± 0.01	9.70	13.60	0.713	0.05	4	1
**B2SD61**	1.27 ± 0.01	8.40	8.90	0.944	0.02	3	3
**B3S15**	0.90 ± 0.01	8.70	8.80	0.989	0.02	1	4
**B3S19**	0.72 ± 0.02	13.80	14.60	0.945	0.08	4	3
**B3S20**	0.75 ± 0.00	6.50	5.90	1.102	0.00	4	4
**B3S22**	0.80 ± 0.02	8.10	10.90	0.743	0.02	3	2
**B3S23**	0.63 ± 0.00	9.80	11.00	0.891	0.03	3	4
**B3S24**	0.05 ± 0.00	14.80	13.00	1.138	0.07	4	4
**B3S31**	0.69 ± 0.02	12.40	14.70	0.844	0.07	3	3
**B3S41**	0.41 ± 0.02	10.30	11.20	0.920	0.03	3	3
**B3S53**	0.51 ± 0.02	11.20	12.50	0.896	0.05	1	1
**B3S57**	0.33 ± 0.00	9.00	8.70	1.034	0.02	4	2
**B3S63**	1.17 ± 0.01	8.10	8.90	0.910	0.02	3	4
**B3S66**	1.19 ± 0.00	11.50	10.00	1.150	0.03	4	3
**B3S7**	0.34 ± 0.00	9.50	7.80	1.218	0.01	4	3

**B3SD10b**	0.71 ± 0.00	14.80	14.90	0.993	0.09	4	3
**B3SD12**	0.60 ± 0.01	16.60	21.50	0.772	0.21	5	3
**B3SD13**	0.47 ± 0.00	14.80	16.30	0.908	0.11	3	3
**B3SD25**	0.57 ± 0.00	11.20	9.80	1.143	0.03	3	3
**B3SD38**	1.03 ± 0.02	9.90	10.90	0.908	0.03	3	3
**B3SD40**	0.61 ± 0.00	8.70	7.80	1.12	0.01	3	1
**B3SD46**	1.03 ± 0.00	7.20	7.10	1.014	0.01	4	4
**B3SD6**	0.33 ± 0.00	9.20	8.10	1.136	0.02	1	4
**B3SD61**	0.56 ± 0.02	10.90	10.10	1.079	0.03	4	3
**B3SD9**	0.48 ± 0.01	10.50	10.60	0.991	0.03	1	1
**B4C1**	0.32 ± 0.01	10.20	9.50	1.074	0.02	3	3
**B4C2**	1.22 ± 0.01	9.70	8.30	1.169	0.02	3	3
**B4C4**	1.15 ± 0.01	13.60	14.80	0.919	0.08	3	4
**B4S16**	0.62 ± 0.00	13.10	15.30	0.856	0.08	4	3
**B4S21**	1.42 ± 0.00	9.30	11.50	0.809	0.03	1	3
**B4S22**	0.79 ± 0.00	8.20	10.30	0.796	0.02	1	1
**B4S23**	0.86 ± 0.01	10.90	12.00	0.908	0.04	4	4
**B4S24**	0.81 ± 0.02	12.10	15.20	0.796	0.08	4	2
**B4S31**	0.01 ± 0.01	16.10	15.40	1.045	0.10	3	2
**B4S33**	0.46 ± 0.01	11.70	14.00	0.836	0.06	3	3
**B4S55**	0.91 ± 0.01	8.70	6.80	1.279	0.01	1	4
**B4S57**	0.49 ± 0.01	12.20	13.40	0.910	0.06	5	3

**B4S58a**	0.78 ± 0.01	9.30	9.50	0.979	0.02	4	3
**B4S63**	1.00 ± 0.01	10.30	11.10	0.928	0.03	1	3
**B4S64**	0.60 ± 0.01	9.90	9.90	1.000	0.03	4	3
**B4SD25**	0.42 ± 0.01	14.80	18.80	0.787	0.14	1	3
**B4SD40**	0.53 ± 0.01	13.40	15.40	0.870	0.09	4	3
**B4SD6**	1.03 ± 0.01	11.10	11.90	0.933	0.04	3	4
**B5C2**	1.09 ± 0.01	9.90	12.30	0.805	0.04	1	1
**B5S1**	0.43 ± 0.01	10.40	10.40	1.000	0.03	1	1
**B5S16**	1.09 ± 0.01	12.00	14.30	0.839	0.07	1	1
**B5S17**	0.65 ± 0.01	12.90	13.60	0.949	0.06	3	4
**B5S18**	1.31 ± 0.01	13.40	16.20	0.83	0.09	4	3
**B5S19**	0.81 ± 0.01	12.00	13.80	0.870	0.06	5	4
**B5S23**	0.61 ± 0.02	10.80	14.70	0.735	0.06	1	1
**B5S28**	1.24 ± 0.01	12.30	10.60	1.160	0.04	4	3
**B5S29**	1.22 ± 0.00	12.30	12.60	0.976	0.05	4	1
**B5S2a**	0.67 ± 0.01	12.40	14.10	0.879	0.07	4	3
**B5S32**	0.86 ± 0.01	15.30	18.70	0.818	0.15	1	2
**B5S36**	1.58 ± 0.01	15.90	21.30	0.746	0.20	6	3
**B5S37**	1.12 ± 0.01	8.40	9.60	0.875	0.02	3	3
**B5S41**	0.28 ± 0.01	9.90	12.90	0.767	0.04	4	2
**B5S43**	0.66 ± 0.01	9.80	12.10	0.810	0.04	4	3
**B5S44**	0.80 ± 0.00	8.70	9.80	0.888	0.02	3	1
**B5S50**	0.57 ± 0.01	13.30	16.20	0.821	0.09	3	1

**B5S51**	0.50 ± 0.01	12.50	12.50	1.000	0.05	3	4
**B5S53**	1.07 ± 0.01	8.30	7.60	1.092	0.01	4	4
**B5S58**	0.71 ± 0.00	12.30	9.40	1.309	0.03	1	3
**B5SD10**	0.73 ± 0.00	12.20	11.80	1.034	0.05	4	4
**B5SD11**	1.16 ± 0.01	10.30	15.00	0.687	0.06	4	2
**B5SD13**	0.76 ± 0.01	7.40	10.00	0.740	0.02	1	1
**B5SD38**	0.02 ± 0.00	12.40	14.80	0.838	0.07	4	3
**B5SD5**	0.66 ± 0.00	14.60	11.30	1.292	0.05	3	3
**B5SD6**	1.02 ± 0.02	8.40	9.40	0.894	0.02	4	3
**B6C2**	1.06 ± 0.01	8.90	9.90	0.899	0.02	3	3
**B6S16**	0.86 ± 0.02	10.00	11.40	0.877	0.03	1	4
**B6S20**	0.85 ± 0.01	11.10	12.50	0.888	0.05	3	3
**B6S22**	0.22 ± 0.00	9.10	10.80	0.843	0.03	3	3
**B6S23**	1.08 ± 0.01	14.10	18.90	0.746	0.14	3	4
**B6S24**	0.51 ± 0.01	14.00	14.20	0.986	0.08	4	3
**B6S28**	0.43 ± 0.01	8.40	7.50	1.120	0.01	1	4
**B6S29**	0.81 ± 0.01	10.40	10.80	0.963	0.03	3	4
**B6S33**	1.44 ± 0.00	9.00	11.50	0.783	0.03	3	1
**B6S35**	0.94 ± 0.00	8.00	7.00	1.143	0.01	3	3
**B6S36**	0.25 ± 0.00	11.00	12.00	0.917	0.04	3	4
**B6S43**	1.14 ± 0.01	14.10	20.50	0.688	0.16	1	3
**B6S49**	0.84 ± 0.01	11.20	10.30	1.087	0.03	4	4

**B6S52**	0.92 ± 0.01	10.10	11.80	0.856	0.04	4	4
**B6S53**	1.29 ± 0.02	10.40	13.30	0.782	0.05	4	4
**B6S55**	0.78 ± 0.01	7.90	9.30	0.849	0.02	1	3
**B6S56**	0.87 ± 0.02	7.40	8.30	0.892	0.01	1	4
**B6S57**	0.72 ± 0.01	9.30	9.20	1.011	0.02	1	1
**B6S58**	0.12 ± 0.00	9.20	11.30	0.814	0.03	3	3
**B6S64**	0.54 ± 0.00	12.10	15.50	0.781	0.08	4	2
**B6SD12**	1.50 ± 0.01	12.00	14.40	0.833	0.07	3	3
**B6SD13**	0.84 ± 0.01	9.10	7.30	1.247	0.01	1	4
**B6SD14**	0.26 ± 0.01	7.90	8.20	0.963	0.01	1	1
**B6SD6**	0.29 ± 0.02	14.40	18.40	0.783	0.13	5	3
**B6SD61**	0.64 ± 0.02	11.20	10.40	1.077	0.03	3	3
**B6SD9**	0.61 ± 0.01	12.10	13.90	0.871	0.06	1	2
**B7C1**	0.07 ± 0.01	9.70	7.30	1.329	0.01	1	4
**B7C4a**	1.26 ± 0.01	11.80	8.40	1.405	0.02	3	1
**B7S16**	0.82 ± 0.01	10.30	13.00	0.792	0.05	4	4
**B7S17**	1.40 ± 0.01	15.2	16.4	0.927	0.11	4	3
**B7S2**	1.02 ± 0.01	11.10	9.70	1.144	0.03	1	3
**B7S21**	0.25 ± 0.00	12.00	17.60	0.682	0.10	3	3
**B7S28**	1.04 ± 0.01	16.30	17.00	0.959	0.13	5	3
**B7S29**	1.15 ± 0.02	7.70	7.90	0.975	0.01	2	4
**B7S32**	1.33 ± 0.01	11.60	14.10	0.823	0.06	4	4
**B7S33**	1.15 ± 0.01	10.70	10.90	0.982	0.03	3	3

**B7S34**	0.52 ± 0.00	13.40	15.20	0.882	0.08	4	4
**B7S35**	0.50 ± 0.02	8.10	9.30	0.871	0.02	3	1
**B7S49**	0.67 ± 0.00	12.50	13.20	0.947	0.06	4	4
**B7S54b**	1.05 ± 0.02	12.10	17.40	0.695	0.10	4	3
**B7S66**	0.83 ± 0.01	9.60	8.80	1.091	0.02	3	3
**B7S7**	1.46 ± 0.01	12.80	17.70	0.723	0.11	1	1
**B7S8**	0.78 ± 0.01	15.30	21.60	0.708	0.19	4	3
**B7SD11**	1.10 ± 0.01	11.30	12.40	0.911	0.05	2	2
**B7SD25**	0.69 ± 0.01	12.30	11.10	1.108	0.04	4	4
**B7SD38**	1.24 ± 0.00	15.20	16.20	0.938	0.11	4	4
**B7SD46**	0.78 ± 0.01	8.40	11.10	0.757	0.03	3	1
**B7SD6**	0.64 ± 0.02	13.60	16.40	0.829	0.10	3	3
**B7SD9**	0.44 ± 0.01	13.40	12.90	1.039	0.06	3	4
**B8C1**	0.12 ± 0.01	12.10	11.90	1.017	0.05	3	3
**B8C4a**	0.77 ± 0.01	11.00	11.00	1.000	0.04	3	3
**B8S1**	0.64 ± 0.01	13.70	12.80	1.070	0.06	1	3
**B8SD14**	0.91 ± 0.01	11.00	12.70	0.866	0.05	3	3
**B8S16**	0.66 ± 0.01	9.20	10.60	0.868	0.03	3	3
**B8S17**	0.72 ± 0.01	13.00	11.20	1.161	0.04	4	3
**B8S18**	1.04 ± 0.01	10.70	9.70	1.103	0.03	4	1
**B8S19**	0.59 ± 0.01	11.50	14.40	0.799	0.06	1	3
**B8S2**	0.74 ± 0.00	12.80	14.90	0.859	0.08	3	3

**B8S20**	1.18 ± 0.01	11.60	12.90	0.899	0.05	3	4
**B8S21**	1.00 ± 0.01	12.20	13.10	0.931	0.06	3	3
**B8S22**	0.91 ± 0.00	8.60	8.80	0.977	0.02	3	2
**B8S23**	1.21 ± 0.01	10.00	10.60	0.943	0.03	1	1
**B8S24**	1.28 ± 0.01	16.30	20.80	0.784	0.19	4	3
**B8S29**	0.55 ± 0.01	6.50	9.30	0.699	0.01	4	1
**B8S3**	0.43 ± 0.01	11.50	11.80	0.975	0.04	3	1
**B8S31**	0.50 ± 0.02	14.80	17.10	0.865	0.12	4	4
**B8S32**	0.60 ± 0.01	11.20	11.20	1.000	0.04	3	4
**B8S33**	1.04 ± 0.01	8.50	10.90	0.780	0.03	4	1
**B8S35**	0.84 ± 0.01	9.00	9.20	0.98	0.02	1	4
**B8S36**	0.45 ± 0.01	14.10	14.20	0.993	0.08	3	3
**B8S37**	0.72 ± 0.02	10.10	11.50	0.878	0.04	3	3
**B8S42**	0.77 ± 0.01	9.60	11.70	0.821	0.03	3	1
**B8S43**	1.00 ± 0.01	10.20	8.70	1.172	0.02	4	4
**B8S49**	0.28 ± 0.01	13.80	14.60	0.945	0.08	4	4
**B8S51**	1.41 ± 0.01	10.50	10.50	1.000	0.03	1	3
**B8S52**	0.81 ± 0.01	10.50	12.20	0.861	0.04	3	4
**B8S54**	1.25 ± 0.02	10.40	13.30	0.782	0.05	4	2
**B8S55**	1.00 ± 0.01	8.50	10.00	0.850	0.02	4	1
**B8S58**	1.00 ± 0.01	12.50	15.40	0.812	0.08	3	3
**B8S63**	0.87 ± 0.01	9.20	10.80	0.852	0.03	4	1
**B8S64**	0.79 ± 0.01	11.60	14.90	0.779	0.07	3	3

**B8SD11**	0.79 ± 0.01	11.30	12.90	0.876	0.05	3	4
**B8SD25**	1.07 ± 0.01	10.40	9.90	1.051	0.03	1	3
**B8SD40**	0.52 ± 0.00	9.20	9.20	1.000	0.02	3	2
**B8SD5**	0.92 ± 0.01	15.90	15.90	1.000	0.11	3	4
**B9S15**	0.94 ± 0.01	11.80	15.30	0.771	0.07	4	4
**B9S16**	0.73 ± 0.02	17.00	16.90	1.006	0.13	4	4
**B9S19**	0.66 ± 0.01	11.10	12.40	0.895	0.05	3	3
**B9S22**	0.04 ± 0.01	14.20	17.30	0.821	0.11	1	1
**B9S23**	0.45 ± 0.02	10.00	9.80	1.020	0.02	4	4
**B9S27**	0.96 ± 0.00	8.60	7.70	1.117	0.01	3	1
**B9S32**	0.41 ± 0.01	8.40	9.20	0.913	0.02	4	3
**B9S33**	0.10 ± 0.01	10.00	10.10	0.990	0.03	4	3
**B9S34**	0.29 ± 0.01	8.60	6.90	1.246	0.01	3	1
**B9S41**	0.68 ± 0.01	14.10	16.00	0.881	0.10	4	4
**B9S42**	0.98 ± 0.00	8.90	9.30	0.957	0.02	4	1
**B9S43**	0.74 ± 0.01	10.60	11.30	0.938	0.04	1	1
**B9S50**	0.17 ± 0.00	10.30	11.10	0.928	0.03	1	1
**B9S53**	0.70 ± 0.01	11.40	13.10	0.870	0.05	4	4
**B9S54**	0.81 ± 0.01	13.50	18.80	0.718	0.13	3	4
**B9S55**	0.77 ± 0.01	11.80	14.20	0.831	0.06	4	3
**B9S56**	0.20 ± 0.02	11.00	11.40	0.965	0.04	1	3
**B9S58**	0.41 ± 0.01	12.70	13.40	0.948	0.06	4	3

**B9S62**	0.49 ± 0.02	11.00	9.70	1.134	0.03	4	4
**B9S8**	0.67 ± 0.01	14.40	14.70	0.980	0.08	4	4
**B9SD10**	1.28 ± 0.00	11.00	13.40	0.821	0.05	4	3
**B9SD12**	0.89 ± 0.01	11.20	13.90	0.806	0.06	4	4
**B9SD13**	0.70 ± 0.01	12.20	14.60	0.836	0.07	3	3
**B9SD25**	0.70 ± 0.01	11.40	12.40	0.919	0.05	2	2
**B9SD38**	0.84 ± 0.01	11.30	13.90	0.813	0.06	4	3
**B9SD40**	1.03 ± 0.02	10.50	12.50	0.840	0.04	1	1
**B9SD5**	0.71 ± 0.01	11.90	12.40	0.960	0.05	3	1
**B9SD6**	0.58 ± 0.01	13.10	17.20	0.762	0.10	1	3
**B9SD60**	0.51 ± 0.02	14.60	14.60	1.000	0.08	3	4
**B9SD9**	0.92 ± 0.01	10.30	10.80	0.954	0.03	3	2
**B13C2**	0.82 ± 0.01	13.80	18.10	0.762	0.12	3	2
**B13C4a**	0.90 ± 0.01	9.40	7.70	1.221	0.01	1	4
**B13C4b**	1.37 ± 0.02	8.60	8.30	1.036	0.01	1	1
**B13S15**	0.72 ± 0.01	12.20	13.60	0.897	0.06	4	4
**B13S17**	0.80 ± 0.01	7.00	9.30	0.753	0.02	3	3
**B13S18**	0.70 ± 0.01	7.10	9.80	0.724	0.02	4	1
**B13S19**	1.15 ± 0.02	13.40	15.60	0.859	0.09	4	3
**B13S20**	0.76 ± 0.01	8.80	10.90	0.807	0.03	3	1
**B13S21**	0.12 ± 0.01	12.30	13.00	0.946	0.06	4	4
**B13S23**	0.86 ± 0.01	10.20	13.00	0.785	0.05	1	3
**B13S24**	1.03 ± 0.00	12.50	14.80	0.845	0.07	4	4

**B13S27**	1.39 ± 0.01	11.20	16.20	0.691	0.08	1	1
**B13S28**	1.12 ± 0.01	15.50	18.00	0.861	0.14	1	2
**B13S29**	0.25 ± 0.01	12.30	12.30	1.000	0.05	3	3
**B13S3**	0.68 ± 0.01	13.20	13.60	0.971	0.07	3	2
**B13S30**	0.30 ± 0.01	12.70	14.80	0.858	0.07	4	3
**B13S31**	0.96 ± 0.00	9.10	6.90	1.319	0.01	1	1
**B13S33**	0.68 ± 0.01	9.10	12.40	0.734	0.04	4	3
**B13S34**	0.28 ± 0.01	3.60	4.30	0.837	0.00	1	1
**B13S35**	0.81 ± 0.01	13.30	18.30	0.727	0.12	1	3
**B13S37**	0.82 ± 0.01	11.30	12.10	0.934	0.04	4	1
**B13S39**	0.98 ± 0.01	9.30	13.00	0.715	0.04	3	1
**B13S41**	1.08 ± 0.02	13.20	14.20	0.930	0.07	3	2
**B13S43**	1.21 ± 0.02	10.80	13.30	0.812	0.05	3	3
**B13S44**	0.46 ± 0.00	14.20	16.20	0.877	0.10	4	1
**B13S45**	0.95 ± 0.00	8.20	9.90	0.828	0.02	4	4
**B13S51**	0.54 ± 0.01	17.40	20.90	0.833	0.21	1	4
**B13S53**	1.32 ± 0.02	10.60	13.20	0.803	0.05	4	3
**B13S55**	0.82 ± 0.01	11.10	12.40	0.895	0.05	2	3
**B13S56**	0.87 ± 0.00	10.30	12.10	0.851	0.04	3	3
**B13S57**	0.06 ± 0.01	9.70	10.80	0.898	0.03	3	1
**B13S58**	0.38 ± 0.01	12.60	14.10	0.894	0.07	3	3
**B13S62**	1.55 ± 0.02	11.20	11.50	0.974	0.04	4	4

**B13S63**	0.65 ± 0.01	11.70	13.70	0.854	0.06	4	4
**B13S64**	0.67 ± 0.01	12.30	13.50	0.911	0.06	1	1
**B13S65**	0.42 ± 0.01	11.80	13.40	0.881	0.06	3	4
**B13S66**	0.86 ± 0.01	10.00	9.00	1.111	0.02	3	4
**B13SD10**	1.13 ± 0.01	12.70	14.80	0.858	0.07	4	3
**B13SD11**	0.47 ± 0.01	10.40	12.00	0.867	0.04	3	2
**B13SD12**	0.79 ± 0.01	10.60	15.10	0.702	0.06	1	2
**B13SD14**	0.66 ± 0.00	9.50	9.00	1.056	0.02	4	4
**B13SD38**	0.79 ± 0.01	10.40	14.20	0.732	0.06	3	2
**B13SD4**	0.91 ± 0.01	10.40	9.30	1.118	0.02	3	3
**B13SD40**	1.31 ± 0.01	6.90	7.80	0.885	0.01	3	1
**B13SD46**	1.10 ± 0.01	9.20	9.80	0.939	0.02	1	4
**B13SD5**	0.70 ± 0.00	15.10	16.40	0.921	0.11	5	3
**B13SD6**	0.24 ± 0.01	8.40	7.80	1.077	0.01	1	1
**B13SD60**	0.49 ± 0.01	15.60	20.80	0.750	0.18	4	4
**B13SD61**	0.96 ± 0.01	8.60	11.50	0.748	0.03	3	1
**B13SD9**	0.02 ± 0.01	11.90	13.10	0.908	0.05	4	3
**B14S17**	0.86 ± 0.02	9.00	10.90	0.826	0.03	4	1
**B14S18**	0.61 ± 0.01	11.30	10.40	1.087	0.03	4	3
**B14S2**	0.91 ± 0.01	10.80	11.70	0.923	0.04	1	1
**B14S24**	0.47 ± 0.01	14.20	13.60	1.044	0.07	4	4
**B14S27**	1.17 ± 0.01	11.30	11.80	0.958	0.04	3	1
**B14S29**	1.47 ± 0.01	9.80	9.90	0.990	0.02	1	1

**B14S31**	0.68 ± 0.02	7.40	5.40	1.370	0.00	1	1
**B14S34**	1.15 ± 0.01	10.30	13.00	0.792	0.05	4	4
**B14S37**	0.12 ± 0.01	11.00	13.30	0.827	0.05	4	3
**B14S44**	0.46 ± 0.00	11.90	12.50	0.952	0.05	3	3
**B14S45**	1.08 ± 0.00	12.20	15.70	0.777	0.08	4	4
**B14S51**	1.41 ± 0.00	7.90	6.50	1.215	0.01	2	4
**B14S52**	0.14 ± 0.01	10.10	14.50	0.697	0.06	1	1
**B14S55**	0.25 ± 0.01	10.10	15.30	0.660	0.06	4	1
**B14S56**	0.62 ± 0.01	7.10	7.90	0.899	0.01	1	1
**B14S58**	0.33 ± 0.02	11.00	11.80	0.932	0.04	4	3
**B14SD10**	0.28 ± 0.01	12.20	13.10	0.931	0.06	2	2
**B14SD11**	0.79 ± 0.01	11.00	12.80	0.859	0.05	4	3
**B14SD13**	0.37 ± 0.01	12.80	15.10	0.848	0.08	1	3
**B14SD14**	0.83 ± 0.01	10.90	11.90	0.916	0.04	4	4
**B14SD5**	0.76 ± 0.01	11.60	11.30	1.027	0.04	1	1
**B15S1**	0.57 ± 0.02	7.40	6.60	1.121	0.01	1	3
**B15S17**	0.53 ± 0.01	8.90	14.40	0.618	0.05	4	1
**B15S27**	0.34 ± 0.01	7.70	7.30	1.055	0.01	4	3
**B15S34**	0.29 ± 0.01	11.40	11.90	0.958	0.04	1	3
**B15S53**	0.68 ± 0.01	10.20	11.20	0.911	0.03	1	1
**B15S63**	0.95 ± 0.01	11.90	14.50	0.821	0.07	4	4
**B15SD11b**	1.04 ± 0.01	9.10	10.80	0.843	0.03	1	3

**B15SD14**	0.92 ± 0.00	7.60	10.30	0.738	0.02	1	1
**B15SD38**	0.28 ± 0.02	12.40	16.50	0.752	0.09	4	4
**B15SD5**	0.73 ± 0.01	6.20	5.80	1.069	0.00	3	3
**B15SD61**	0.87 ± 0.01	10.60	10.50	1.010	0.03	4	4
**B16S53**	0.88 ± 0.01	11.30	10.20	1.108	0.03	4	4
**B16SD5**	0.42 ± 0.01	15.30	14.10	1.085	0.08	4	3

Quantitative traits: Oil yield: mean essential oil content (± standard deviation of each individual) expressed as a percentage of *mL* of essential oil per *g* of leaf fresh weight (% v/fw); TH: tree height; DBH: diameter at breast height; SC: slenderness coefficient; VOL: wood volume. **Categorical traits**: ST: stem straightness; BQ: branching quality.

Supplementary Table 2. Statistical parameters associated with the measurement of quantitative traits (Oil yield, TH, DBH, VOL, and SC) and categorical traits (ST and BQ) from 339 *E. globulus* individuals randomly selected from the breeding population.

**Statistical parameter**	**Oil yield (% v/fw)**	**TH (m)**	**DBH (cm)**	**VOL (m^3)**	**SC (TH/DBH)**	**ST**	**BQ**
Mean	0.78	11.02	12.36	0.91	0.05	2.93	2.65
Median	0.78	10.90	12.20	0.89	0.04	3.00	3.00
Mode	0.81	10.30	13.00	1.00	0.02	4.00	3.00
Range	0.01-1.82	3.60-18.00	4.30-22.70	0.60-1.77	0.01-0.22	1.00-6.00	1.00-4.00
SD	0.35	2.29	3.25	0.15	0.04	1.21	1.13

Oil yield: essential oil content expressed as a percentage of *mL* of essential oil per *g* of leaf fresh weight (% v/fw); TH: tree height; DBH: diameter at breast height; SC: slenderness coefficient; VOL: wood volume; ST: stem straightness; BQ: branching quality; SD: standard deviation.

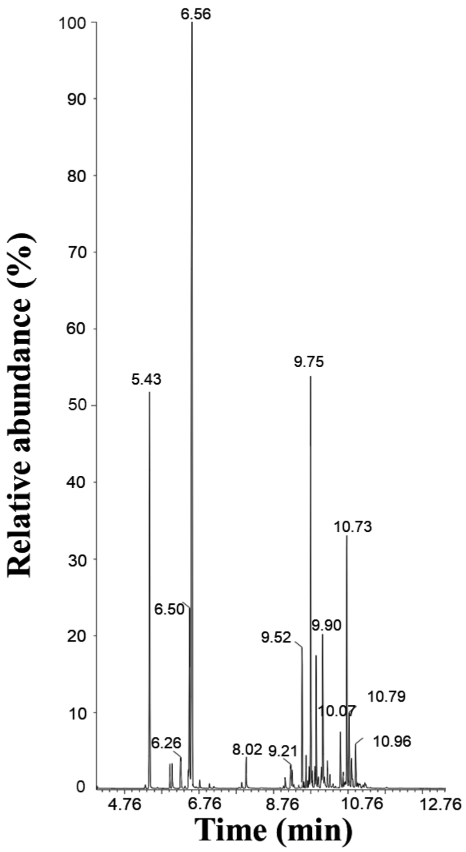

Peak identification

- 5.43 min: α-Pinene (52%)
- 6.50 min: D-Limonene (23%)
- 6.57 min: 1,8-cineole (100%)
- 8.03 min: α-Terpineol (5%)
- 9.07 min: α-Terpineol acetate (32%)
- 9.52 min: α-Gurjunene (18%)
- 9.75 min: 1H-Cycloprop[e]azulene (53%)
- 9.90 min: Alloaromadendrene (21%)
- 10.08 min: (+)-Ledene (7%)
- 10.73 min: Globulol (35%)
- 10.79 min: Viridiflorol (11%)
- 10.96 min: β-Eudesmol (4%)

Supplementary Figure 1. Representative GC-MS chromatogram and NIST identification of main terpenes present in the essential oil of *Eucalyptus globulus* grown in southern Chile. Relative abundance (%) was expressed according to the most intense signal (1,8-cineole).
